# Supplementary material for: Cytokine profiles of mild-to-moderate SARS-CoV-2 infected and recovered pre-vaccinated individuals residing in Indonesia
Source: PeerJ. 2024 Apr 18;12:e17257. doi: 10.7717/peerj.17257 (PMC11032655; doi:10.7717/peerj.17257)
Supplement: Data S2 [file peerj-12-17257-s002.pdf]

Data Interpretation Report

Report Date: 11/17/2022 Report Time: 3:05:27PM

**Batch Name:**	Uji Covid BCA-1	**Serial Number:**	MAGPX13032703
**Protocol Name:**	HCYP2MAG	**Batch Run Date:**	11/17/2022 3:00:55PM
**Protocol Version:**	1	**Operator:**	
**Test Name:**	HBCA-1	**Report Generated By:**	

Formula: Y=a + ((b-a) / (1 + ((x / c) ^ d)) ^ f) (Logistic 5P Weighted)

Most recent calibration and verification results:

**CAL:**	Passed	11/16/2022 2:29:06PM
**VER:**	Passed	11/16/2022 2:30:05PM
**Fluidics:**	Passed	11/16/2022 2:31:19PM

Calibration results used for batch:

**Date:**	11/16/2022 2:29:06PM	**Lot:**	B90644	**Lot Expiration:**	9/22/2023
**CL1 Current:**	259	**CL1 Temp:**	25.80	**CL1 Factor:**	0.00694
**CL2 Current:**	274	**CL2 Temp:**	25.80	**CL2 Factor:**	0.00693
**RP1 Long Current:**	389	**RP1 Long Temp:**	25.80	**RP1 Long Factor:**	0.00384
**RP1 Short Current:**	389	**RP1 Short Temp:**	25.80	**RP1 Short Factor:**	0.11435

Data Interpretation Report

Report Date: 11/17/2022 Report Time: 3:05:27PM

Batch Name: Uji Covid BCA-1
Protocol Name: HCYP2MAG
Protocol Version: 1
Test Name: HBCA-1

Serial Number: MAGPX13032703
Batch Run Date: 11/17/2022 3:00:55PM
Operator:
Report Generated By:

HBCA-1
Logistic5p: y = a+((b-a)/(1+((x/c)^d))^f)

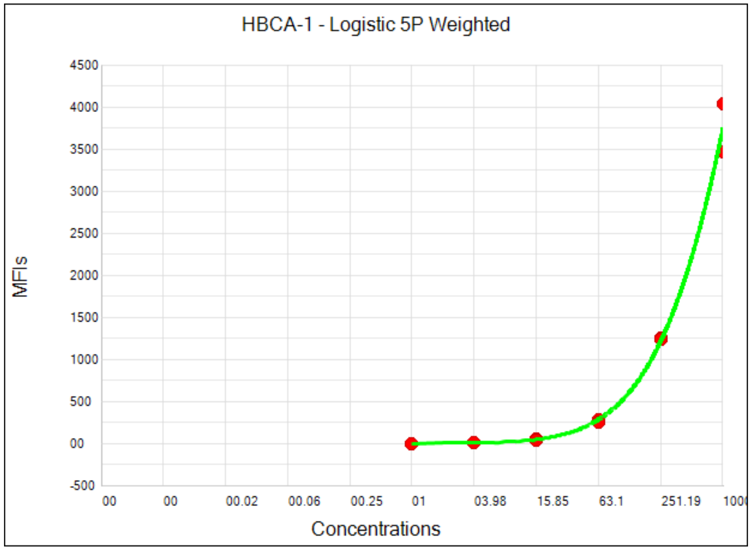

Curve Data:

R^2 = 0.94743596094873

Sum of Residuals = 86.8868861516936

Average Residuals = 7.24057384597446

Relative Sum of Squares Absolute = 168147.980890031

Relative Sum of Squares Relative = 3.26330035528418

Standard Error of the Estimate = 0.682777976388076

Adjusted Coefficient of Multiple Determination = 0.917399367205147

Data Interpretation Report

Report Date: 11/17/2022 Report Time: 3:05:27PM

Batch Name: Uji Covid BCA-1
Protocol Name: HCYP2MAG
Protocol Version: 1
Test Name: HBCA-1

Serial Number: MAGPX13032703
Batch Run Date: 11/17/2022 3:00:55PM
Operator:
Report Generated By:

Coefficient a = -0.976077848060167

Coefficient b = 57034.9271897174

Coefficient c = 63.3958430146589

Coefficient d = -0.307877183760497

Coefficient f = 7.64871805893168

Standards:

Loc	Standard	Expected Conc	Net MFI	Test Result	Range	% Recovery	%CV of Replicates	Unit	Comments
1,A2	Standard6	1,000.00	4040	1115.922		111.592		pg/ml	Extrapolated
1,B2	Standard5	250.00	1250	259.814		103.926		pg/ml	
1,C2	Standard4	62.50	282	63.16		101.056		pg/ml	
1,D2	Standard3	15.63	52.5	17.429		111.544		pg/ml	
1,E2	Standard2	3.91	5	4.157		106.417		pg/ml	
1,F2	Standard1	0.98	0	1.451		148.535		pg/ml	
1,A3	Standard6	1,000.00	3467	898.423		89.842		pg/ml	
1,B3	Standard5	250.00	1241	257.797		103.119		pg/ml	
1,C3	Standard4	62.50	256	58.228		93.165		pg/ml	
1,D3	Standard3	15.63	42	14.945		95.648		pg/ml	
1,E3	Standard2	3.91	4	3.721		95.255		pg/ml	
1,F3	Standard1	0.98	-1	<0.977		NA		pg/ml	
	Avg Standard1		-0.5	NA			NaN		
	Avg Standard2		4.5	3.939			7.827		
	Avg Standard3		47.25	16.187			10.85		
	Avg Standard4		269	60.694			5.746		
	Avg Standard5		1245.5	258.806			0.551		
	Avg Standard6		3753.5	1007.172			15.27		

Data Interpretation Report

Report Date: 11/17/2022 Report Time: 3:05:27PM

Batch Name: Uji Covid BCA-1
Protocol Name: HCYP2MAG
Protocol Version: 1
Test Name: HBCA-1

Serial Number: MAGPX13032703
Batch Run Date: 11/17/2022 3:00:55PM
Operator:
Report Generated By:

Controls:

Loc	Control	Expected Conc	Net MFI	Low Limit	High Limit	Test Result	Range	% Recovery	%CV of Replicates	Unit	Dilution Factor	Comments
1,H2	Control1	11.00	26	8.40	18.00	10.869		98.811		pg/ml	1.00	
1,H3	Control2	45.00	166	32.00	67.00	NA	Invalid curve	0		pg/ml	1.00	Invalid curve fit

Data Interpretation Report

Report Date: 11/17/2022 Report Time: 3:05:27PM

Batch Name: Uji Covid BCA-1
Protocol Name: HCYP2MAG
Protocol Version: 1
Test Name: HBCA-1

Serial Number: MAGPX13032703
Batch Run Date: 11/17/2022 3:00:55PM
Operator:
Report Generated By:

Samples:

Loc	Sample ID	Net MFI	Test Result	Range	%CV of Replicates	Unit	Dilution Factor	Comments
1,G2	Background0	-1					0.00	
1,G3	Background0	1					0.00	
1,A4	Unknown1	405	NA	Invalid curve fit		pg/ml	1.00	Invalid curve fit
1,B4	Unknown2	42	NA	Invalid curve fit		pg/ml	1.00	Invalid curve fit
1,C4	Unknown3	923.5	NA	Invalid curve fit		pg/ml	1.00	Invalid curve fit
1,D4	Unknown4	195	46.571			pg/ml	1.00	
1,E4	Unknown5	135	34.84			pg/ml	1.00	
1,F4	Unknown6	88	25.233			pg/ml	1.00	
1,G4	Unknown7	280.5	62.876			pg/ml	1.00	
1,H4	Unknown8	29	11.67			pg/ml	1.00	
1,A5	Unknown9	180	43.673			pg/ml	1.00	
1,B5	Unknown10	239	54.995			pg/ml	1.00	
1,C5	Unknown11	101	27.948			pg/ml	1.00	
1,D5	Unknown12	141	36.033			pg/ml	1.00	
1,E5	Unknown13	101	27.948			pg/ml	1.00	
1,F5	Unknown14	2435.5	564.068			pg/ml	1.00	
1,G5	Unknown15	4166.5	1167.241			pg/ml	1.00	Extrapolated
1,H5	Unknown16	147.5	37.319			pg/ml	1.00	
1,A6	Unknown17	1017	208.886			pg/ml	1.00	
1,B6	Unknown18	410.5	87.499			pg/ml	1.00	
1,C6	Unknown19	191	45.8			pg/ml	1.00	
1,D6	Unknown20	1245	258.693			pg/ml	1.00	
1,E6	Unknown21	664	136.523			pg/ml	1.00	
1,F6	Unknown22	106	28.979			pg/ml	1.00	
1,G6	Unknown23	62	19.591			pg/ml	1.00	
1,H6	Unknown24	412	87.784			pg/ml	1.00	
1,A7	Unknown25	303	67.136			pg/ml	1.00	

Data Interpretation Report

Report Date: 11/17/2022 Report Time: 3:05:27PM

Batch Name: Uji Covid BCA-1
Protocol Name: HCYP2MAG
Protocol Version: 1
Test Name: HBCA-1
Serial Number: MAGPX13032703
Batch Run Date: 11/17/2022 3:00:55PM
Operator:
Report Generated By:

Loc	Sample ID	Net MFI	Test Result	Range	%CV of Replicates	Unit	Dilution Factor	Comments
1,B7	Unknown26	57	18.462			pg/ml	1.00	
1,C7	Unknown27	131	34.042			pg/ml	1.00	
1,D7	Unknown28	62	19.591			pg/ml	1.00	
1,E7	Unknown29	45	15.667			pg/ml	1.00	
1,F7	Unknown30	129.5	33.742			pg/ml	1.00	
1,G7	Unknown31	225	52.325			pg/ml	1.00	
1,H7	Unknown32	80	23.531			pg/ml	1.00	
1,A8	Unknown33	60	19.141			pg/ml	1.00	
1,B8	Unknown34	3409.5	877.905			pg/ml	1.00	
1,C8	Unknown35	68	20.924			pg/ml	1.00	
1,D8	Unknown36	253	57.658			pg/ml	1.00	
1,E8	Unknown37	235	54.233			pg/ml	1.00	
1,F8	Unknown38	566	117.335			pg/ml	1.00	
1,G8	Unknown39	92	26.074			pg/ml	1.00	
1,H8	Unknown40	35	13.216			pg/ml	1.00	
1,A9	Unknown41	99	27.534			pg/ml	1.00	
1,B9	Unknown42	89	25.444			pg/ml	1.00	
1,C9	Unknown43	64	20.038			pg/ml	1.00	
1,D9	Unknown44	237.5	54.709			pg/ml	1.00	
1,E9	Unknown45	226.5	52.611			pg/ml	1.00	
1,F9	Unknown46	92	26.074			pg/ml	1.00	
1,G9	Unknown47	845	172.947			pg/ml	1.00	
1,H9	Unknown48	144	36.628			pg/ml	1.00	
1,A10	Unknown49	122	32.234			pg/ml	1.00	
1,B10	Unknown50	182	44.06			pg/ml	1.00	
1,C10	Unknown51	118	31.426			pg/ml	1.00	
1,D10	Unknown52	777	159.104			pg/ml	1.00	
1,E10	Unknown53	317	69.785			pg/ml	1.00	

Data Interpretation Report

Report Date: 11/17/2022 Report Time: 3:05:27PM

Batch Name: Uji Covid BCA-1
Protocol Name: HCYP2MAG
Protocol Version: 1
Test Name: HBCA-1

Serial Number: MAGPX13032703
Batch Run Date: 11/17/2022 3:00:55PM
Operator:
Report Generated By:

Loc	Sample ID	Net MFI	Test Result	Range	%CV of Replicates	Unit	Dilution Factor	Comments
1,F10	Unknown54	116.5	31.122			pg/ml	1.00	
1,G10	Unknown55	180	43.673			pg/ml	1.00	
1,H10	Unknown56	138	35.437			pg/ml	1.00	
1,A11	Unknown57	63	19.815			pg/ml	1.00	
1,B11	Unknown58	36	13.467			pg/ml	1.00	
1,C11	Unknown59	2422	560.145			pg/ml	1.00	
1,D11	Unknown60	403	86.075			pg/ml	1.00	
1,E11	Unknown61	178	43.285			pg/ml	1.00	
1,F11	Unknown62	124	32.637			pg/ml	1.00	
1,G11	Unknown63	95	26.702			pg/ml	1.00	
1,H11	Unknown64	97.5	27.222			pg/ml	1.00	
1,A12	Unknown65	904.5	185.227			pg/ml	1.00	
1,B12	Unknown66	213	50.029			pg/ml	1.00	
1,C12	Unknown67	0	1.451			pg/ml	1.00	
1,D12	Unknown68	316	69.596			pg/ml	1.00	
1,E12	Unknown69	270	60.886			pg/ml	1.00	
1,F12	Unknown70	76	22.67			pg/ml	1.00	
1,G12	Unknown71	20	9.195			pg/ml	1.00	
1,H12	Unknown72	2661.5	631.416			pg/ml	1.00	
	Avg Background0	0	NA		NaN			
